# A Spanish version of the Three-Dimensional Work Fatigue Inventory (3D-WFI): factor structure, internal consistency, and criterion validity

**DOI:** 10.1186/s12889-024-19120-6

**Published:** 2024-06-16

**Authors:** Emilio Moyano-Díaz, Héctor Vargas-Garrido, Doris Méndez-Campos

**Affiliations:** Faculty of Psychology, University of Talca, Talca, Chile

**Keywords:** Work fatigue, Predictors and consequences of work fatigue, Workers’ health, Quality of life of Chilean workers

## Abstract

**Background:**

In the working population, there are risks of overload due to physical, mental, and emotional demands. No instrument is available in Spanish to measure these three types of work fatigue (WF) separately. This paper adapts the Three-Dimensional Work Fatigue Inventory (3D-WFI) (2015), which is of American origin and measures and differentiates these three different types of WF. It has adequate psychometric properties at its root, as do the subsequent German (2018), Lebanese (2022), and Chinese (2023) adaptations.

**Methods:**

A total of 1100 workers (average age = 40 years) from economic sectors such as security and transportation of valuables, secondary and university educational institutions, and healthcare centers participated. They responded to the 3D-WFI, the Health-Related Quality of Life Index, and the Individual Strength Checklist for concurrent validity effects, together with items with sociodemographic and lifestyle variables.

**Results:**

A confirmatory factor analysis with the total sample 3D-WFI supports its three-dimensionality; Cronbach’s alpha and Omega values are adequate by dimensions: for physical work fatigue (α = 0.92, Ω = 0.92), for mental work fatigue (α = 0.94. Ω = 0.94), and emotional work fatigue (α = 0.95, Ω = 0.95). The 3D-WFI correlates significantly with the Checklist Individual Strength (0.743), and a pattern of significant relationships is found between WF and antecedent variables (e.g., being exposed to heat and noise, emotional labor, concentration, and workwear), as well as some consequences of WF (for example, mental health, absenteeism, work satisfaction, and sleep quality).

**Conclusions:**

We contribute here to the cross-cultural validity of the 3D-WFI, which can be used reliably and validly in the Chilean and probably Latin American working population. Some WF predictor variables are confirmed, as well as WF impacts on the absenteeism, health, and quality of life among workers.

## Background

Fatigue affects between 10% and 33% of the world’s population and occurs most frequently in patients with depression (18.5%), being ranked second among the main complaints of most patients attending a general practitioner [1]. Haro et al. [2] report that around 78% of the workforce, at least once in their lives, will have suffered fatigue in any of its dimensions and that there is a direct relationship between work fatigue (WF) and workplace accidents.

Conceptually, until the 1990s, fatigue was defined as a weariness or tiredness that is externalized in bodily or mental spheres [3], with its origin in work and personal conditions. However, nowadays, probably because work is increasingly more mental than physical and, more particularly, it requires interpersonal relationships (service companies), the emotional component has been added to the construct. Thus, three types of fatigue are distinguished: physical, mental, and emotional [4].

Among the factors identified as causing WF are shift work—with its severe implications for performance and occurrence of accidents at work [5]—reduced rest times, time pressure to meet objectives, adverse weather conditions [6], work overload (particularly cognitive), and some specific occupations such as train operators [7]. Other variables include sustained work stress due to repetitive tasks, continuous muscular demands, and physical-environmental and ergonomic conditions such as vibrations, inadequate lighting, noise, uncomfortable temperature, or high levels of comfort. These factors are considered to represent a risk both for health [8, 9] and for workers’ safety [5, 7] since they can generate subjective, psychophysiological, and behavioral reactions [10], which are compatible with the model indicating that fatigue generates changes in the bodies, performance, and subjective perception of people [9]. A condition that is gaining increasing interest in WF research is sleep disturbance. There is a condition called ‘shift work sleep disorder’ (SWSD) [11], characterized by excessive sleepiness or temporary insomnia [12], and that condition is critical in Chile, where sleeping time (on average 7.2 h) is lower than in other countries (for example, Japan with 7.5 h or the Netherlands with slightly more than 8 h) [13]. Therefore, a comprehensive understanding of WF requires the analysis of four sets of factors: those related to work (workload, level of effort, duration, and work schedule); individual factors (age, personality, physical condition, health, diet, consumption of medications, and level of experience); environmental factors (monotony of the environment, noise or vibration levels, temperature and lighting, workplace ergonomics, and tools used for the job); and finally, social factors (lifestyle and quality of life, work conditions, economic precariousness, fear, anxiety, stress, social and family responsibilities) [14].

### The three-dimensional work fatigue inventory (3D-WFI) (Frone & Tidwell, 2015)

In 2015, Frone and Tidwell, in a conceptual and instrumental review of WF, distinguished three different types of WF, namely physical, mental, and emotional, and through two empirical psychometric studies gave rise to the Three-Dimensional Work Fatigue Inventory (3D-WFI) [4]. In a first pilot study with 207 adult participants aged 18 to 65 in different states of the USA (62% women), a tri-factor structure of the instrument was verified (three dimensions of six items each), explaining 78% of the total variance. Alpha coefficients were 0.94 for physical, 0.95 for mental, and 0.96 for emotional fatigue. The lowest inter-item correlation was 0.62 for physical fatigue items, followed by 0.67 for mental fatigue items, and 0.72 for emotional fatigue items. A second study with a sample of 2,477 US workers, via telephone application of the instrument, provided confirmatory evidence of the psychometric results of the first study. The factorial structure of the instrument was tested by verifying three models of one, two, and three factors, providing evidence of the validity of the instrument by confirming numerous hypotheses of the relationship between WF and job demands, job resources, and personality, and with outcome variables or effects (p. 283) [4]. A confirmatory factor analysis (CFA) supported the psychometric properties of the 3D-WFI, adequately discriminating items in each type of fatigue. Convergent and discriminant validity were provided with specific relationship patterns between types of job demands and WF. Emotional demands predicted not only high levels of emotional WF but also high levels of physical WF and mental WF, suggesting that emotional job demands may deplete all energy resources more than physical or mental demands.

Research has indicated that the 3D-WFI may predict important outcomes such as job performance, health outcomes, and workplace safety incidents [15, 16]. The evidence highlights that 3D-WFI has cross-cultural validity, making it a valuable tool for researchers and practitioners in organizational settings. It has been translated, adapted, and validated in Germany [15] in 439 workers (average age = 39.2 years, mostly women, 73%) while also reaching high levels of reliability, alpha = 0.93 for physical fatigue, 0.94 for mental fatigue, and 0.96 for emotional fatigue (p. 677), and validity. The instrument was also adapted and applied in Lebanon to 401 physicians and advanced medical students, with an average age of 34.5 years (57.9% women) [17], and to 435 pharmaceutical chemists (52% men, with an average age of 38.9 years), mostly pharmacy owners, who worked more than 40 h per week [18], while also achieving adequate psychometric indices of adequate construct validity. Likewise, a Brazilian (Portuguese) adaptation has also shown a good level of internal consistency (α = 0.95 and ω = 0.97), with its three dimensions explaining 62.77% of the variance, using a sample of 318 nursing professors from Brazilian federal and state public universities [16]. Most recently, it showed adequate structural validity in a sample of Chinese teachers (overall α = 0.92) [19].

All in all, the 3D-WFI, in addition to its good psychometric properties, is composed of items written in simple language, with potential for application to the Latin American culture of workers with different educational levels. The purpose here is to translate it and test its application to Chilean workers to establish some of its main psychometric properties and to have an adequate instrument to measure WF in the country and probably for Latin America.

## Method

This is a cross-sectional and psychometric study of a Chilean adult working population.

### Sample

The sample comprised a total of 1100 workers belonging to the security services sector, two educational institutions (a state university and a secondary school, both with participants from the administrative and academic staff), and a private health center. The average age of the participants was 40 (SD = 11.5; 62% were male and 38% female). All participants agreed to take part voluntarily, and anonymity was assured; they gave informed consent (ethical certification, University of Talca, Folio 43-2021, 15/06/22) and responded to the survey. To encourage participation, the opportunity to enter a raffle to win supermarket gift cards was offered.

### Instruments

Two different instruments were included to measure WF (i, ii) and a third for self-assessment of health (iii), as well as a set of items to record sociodemographic characteristics (iv), which will be used for the analysis of theoretical or nomological validation.

i) The Three-Dimensional Work Fatigue Inventory (3D-WFI) by Frone and Tidwell [4]. This consists of 18 questions divided into three parts of six items each, evaluating physical, mental, and emotional work fatigue, respectively, during the last 12 months. In all three dimensions, work fatigue increases with higher scores. It employs a Likert-type response format with five alternatives (Every day; At least once a week; At least once a month; Less than once a month; and Never).
ii) The Checklist Individual Strength (CIS) of Beurskens, Bültmann, Kant, Vercoulen, Bleijenberg, and Swaen (2000) in its American version of 20 items [20], which are distributed in three dimensions and are answered on a Likert scale with scores from 1 to 7, where 1 is “Yes‚ that is true” and 7 is “No‚ that is not true,” with a total alpha reliability of 0.90. Previous Chilean versions of 15 items include the one by Vera et al. [21], yielding a Cronbach’s alpha reliability of 0.78 and 0.85 (in each subscale), and by Seguel and Valenzuela [22], with an alpha reliability of 0.78.
iii) Health-Related Quality of Life Index (HRQLI), by Hennessy et al. [23], Spanish version [24]. This measures general self-assessment of health by single items with a five-choice response format (Poor, Fair, Good, Very Good, Excellent) and asks for the number of days on which the respondents felt ill in terms of their physical health, and the same for their mental health, and finally, for how many days in the last month they were absent from work due to illness. Its reliability in the Chilean population is 0.66 [24].
iv) A total of 35 items aimed at identifying the daily and weekly type of shift, level of remuneration, daily water and fruit intake habits, rest, possible care of others at home, quality and quantity of sleep, number of workplace or commuting accidents in the last five years, environmental characteristics of the workplace (noise, temperature, or extreme conditions), reports of household chores that may inhibit adequate rest, centrality of life and enjoyment of work, and life satisfaction.

### Procedure

The two instruments used for measuring WF were translated into Spanish independently by two bilingual English-Spanish academics, whose versions the researchers of the present project later contrasted. By agreement, they decided on a version that they considered to be more in line with the original. The aim was to use colloquial language in Chilean Spanish, thus facilitating its understanding according to the educational or cultural differences of the respondents. In carrying out the translation of the CIS, previous Chilean versions were also considered [21, 22].

Secondly, different companies were contacted through the Chilean Safety Association (ACHS, the leading insurer of occupational accidents and diseases in the country). After authorization by managers, workers were invited to participate in an online study to validate an instrument to measure WF.

### Analysis plan

The reliability of the instruments, means, standard deviations per dimension of each instrument, and total values were calculated. Confirmatory factor analysis (CFA) and one and three-factor models were applied to verify the factor structures for the construct validity of the 3D-WFI. To assess the fit of each model, we used the chi-square indicator, the comparative fit index (CFI ≥ 0.90), the Tucker Lewis Index (TLI ≥ 0.90), the Root Mean Square Residual RMSEA (< 0.08), and the Normed Fit Index (NFI ≥ 0.90), following specialized literature [25]. The best model is the one that meets the cut-off points of the fit indicators. Regression analyses of the 3D-WFI scores along with those of the health self-assessment will be analyzed, with negative correlations with each type of fatigue and general health self-assessment being expected. In contributing to the criterion validity of the 3D-WFI, logistic regressions are used (employing each of the three types of fatigue as dependent variables), expecting to find positive relationships with those items referring to conditions that the literature has found to produce WF, including work overload, exposure to high temperature and noise, type of shift work, and workwear, as well as the effects of WF on job satisfaction and quality of sleep. Additionally, to evaluate the validity of the 3D-WFI, we have correlated its results with those of the Checklist Individual Strength (CIS) of Beurskens et al. [20]—the other instrument for measuring work fatigue—expecting to obtain a positive and significant correlation. The SPSS v25 and AMOS v23 programs are used to perform these analyses.

## Results

First, it reports the CFA of the 3D-WFI with its fit indices, the total reliability indices, and the type of fatigue. Second, the means, standard deviations, and factor loadings for each instrument item are reported. Third, regression analysis of work fatigue on predictor variables and, finally, regression of work fatigue on self-assessment of general, physical, and mental health, absenteeism, job satisfaction, sleep quality, work and commuting accidents, and enjoyment of work are reported.

### Confirmatory factor analysis and reliability of the 3D-WFI

**Fig. 1:**
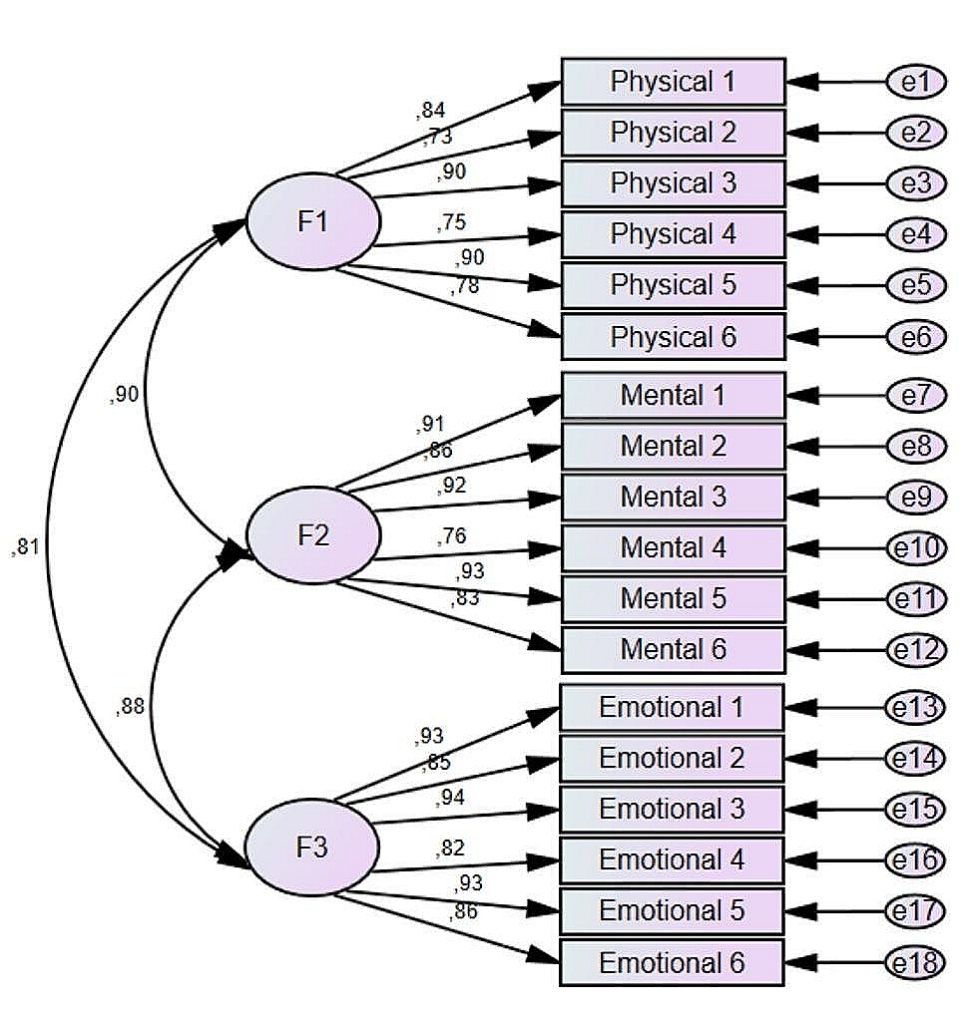
Factors (3) and factor loadings of 3D-WFI (*n* = 1100)

The fit indices of the one-factor model report a poor fit compared to the proposed three-factor model (*χ*2 = 4101.38, *df* = 135, *p* < 0.001, RMSEA = 0.164, CFI = 0.826, TLI = 0.803, NFI = 0.821). The CFA reaffirms the three-factor structure of the 3D-WFI reported by its authors (see Fig. 1). The fit indices are adequate (*χ2* = 1856.66, *df* = 132, *p* < 0.001, RMSEA = 0.109, CFI = 0.924, TLI = 0.912, NFI = 0.919). In turn, the alpha and omega reliability indices for the total scale are high (α = 0.97, Ω = 0.97) and very adequate by dimension: for physical work fatigue (α = 0.92, Ω = 0.92), for mental work fatigue (α = 0.94. Ω = 0.94), and for emotional work fatigue (α = 0.95, Ω = 0.95).

A multigroup confirmatory factor analysis was performed to test the measurement invariance of the 3D-WFI instrument in male and female groups. The metric or weak invariance test, in which the factor loadings (λ) were restricted so that the factor loadings were equal between the groups, showed a good fit, according to the criteria set forth by Cheung & Rensvold [26]. When comparing Model 1 (M1) with Model 2 (M2), no significant changes are presented in the CFI, RMSEA, or χ2 indices. Moreover, both for the scalar invariance model, where factor loadings (λ) and intercepts (τ) are restricted, and for the strict invariance model, where error variances (ɵ) are also restricted, a partial fit is reported since the CFI and RMSEA indices are reported within tolerable ranges. However, the χ2 index reports differences in the measures of the groups (see Table 1).

**Table 1:** Measurement invariance indicators

	χ^2^ (gl)	χ^2^/gl	CFI	RMSEA	Comparison Criteria	Δχ^2^ *p* > 0.05	ΔCFI ≤ 0.01	ΔRMSEA ≤ 0.015
M1. Configuration invariance (baseline)	1999.644 (264)	7.574	0.919	0.081				
M2. Metric or weak invariance (λ constrained)	2016.072 (279)	7.226	0.919	0.079	M2 vs. M1	16.429	2016.072 (279)	7.226
M3 scalar or strong invariance (restricted λ and τ)	2100.985 (297)	7.074	0.916	0.078	M3 vs. M2	84.913	2100.985 (297)	7.074
M4. strict invariance (λ, τ and constrained ɵ)	2184.243 (321)	6.804	0.913	0.076	M4 vs. M3	83.257	2184.243 (321)	6.804

The means, standard deviations, and factor loadings for the instrument (of the Spanish version) are shown in Table 2. For the original English version in the same listing order, see Frone & Tidwell (p. 287) [4].

**Table 2:** Means, standard deviations, and factor loadings for the 3D-WFI items (Spanish version)

3D-WFI Items by Dimensions		Mean (*n* = 1100)	SD	Standardized Factorial Loadings
Physical 1	¿Se sintió físicamente exhausto al final de la jornada de trabajo?	2.70	1.43	0.84
Physical 2	¿Tuvo dificultades para realizar una actividad física al final de la jornada de trabajo?	3.27	1.44	0.73
Physical 3	¿Se sintió físicamente agotado al final de la jornada de trabajo?	2.69	1.29	0.90
Physical 4	¿Quiso desconectarse físicamente al final de la jornada de trabajo?	2.53	1.42	0.75
Physical 5	¿Se sintió físicamente extenuado al final de la jornada de trabajo?	2.89	1.34	0.91
Physical 6	¿Quiso evitar todo aquello que requería demasiada energía física al final de la jornada de trabajo?	3.16	1.46	0.78
	Overall Physical Fatigue	3.14	1.15	
Mental 1	¿Se sintió mentalmente exhausto al final de la jornada de trabajo?	2.86	1.34	0.91
Mental 2	¿Tuvo dificultades para pensar y concentrarse al final de la jornada de trabajo?	3.27	1.42	0.86
Mental 3	¿Se sintió mentalmente agotado al final de la jornada de trabajo?	2.91	1.37	0.92
Mental 4	¿Quiso desconectarse mentalmente al final de la jornada de trabajo?	2.58	1.42	0.77
Mental 5	¿Se sintió mentalmente extenuado al final de la jornada de trabajo?	2.98	1.34	0.93
Mental 6	¿Quiso evitar todo aquello que requería demasiada energía mental al final de la jornada de trabajo?	3.13	1.45	0.83
	Overall Mental Fatigue	3.04	1.23	
Emotional 1	¿Se sintió emocionalmente exhausto al final de la jornada de trabajo?	3.14	1.36	0.93
Emotional 2	¿Tuvo dificultades para mostrar y manejar sus emociones al final de la jornada de trabajo?	3.55	1.37	0.85
Emotional 3	¿Se sintió emocionalmente agotado al final de la jornada de trabajo?	3.20	1.37	0.94
Emotional 4	¿Quiso desconectarse emocionalmente al final de la jornada de trabajo?	2.96	1.47	0.82
Emotional 5	¿Se sintió emocionalmente extenuado al final de la jornada de trabajo?	3.21	1.37	0.94
Emotional 6	¿Quiso evitar todo aquello que requería demasiada energía emocional al final de la jornada de trabajo?	3.31	1.43	0.86
	Overall Emotional Fatigue	2.76	1.27	
	Overall 3D-WFI	2.99	1.14	

A higher average is observed for physical fatigue, followed by mental fatigue, and finally, emotional fatigue. All averages are significantly different from each other (see Table 3).

**Table 3:** Means, standard deviations, and means comparisons of the 3D-WFI by dimensions and overall (*n* = 1100)

Dimension	Mean	SD	Means Comparison values	t	gl	*p*
WF_Phys	3.14	1.15	WF_Physical – WF_Mental	5.18	1139	0.001
WF_Ment	3.04	1.23	WF_Physical – F_Emotional	15.52	1099	0.001
WF_Emot	2.76	1.26	WF_Mental – WF_Emotional	13.50	1099	0.001
Overall	2.99	1.14				

In searching for the content validity of the instrument, the 3D-WFI has been correlated with the CIS 20 (Cronbach’s α = 0.93 here), obtaining a significant Pearson correlation of 0.74 (also, Spearman’s Rho = 0.74, *p* = 0.01).

The results of a regression analysis to identify job variables that can predict work fatigue are shown in Table 4.

**Table 4:** Regression of working demands and job characteristics as predictors of WF dimensions

Working demands or job characteristics.	Standardized Betas on WF Dimensions		
	Physical	Mental	Emotional
In your job, you are exposed to:	*β*	*β*	*β*
Heat and noise most of the time	− 0.10**	− 0.09**	− 0.11**
Repetitive or monotonous tasks	− 0.05**	− 0.07**	− 0.06**
Control emotions	− 0.25**	− 0.28**	− 0.31**
Constant concentration	− 0.13**	− 0.16**	− 0.11**
Taking care of others	− 0.02**	− 0.03**	− 0.03**
Harsh environmental conditions (outdoors, on the street, etc.)	− 0.04**	− 0.07**	− 0.01**
Wearing uniforms, suits, or specific clothing.	− 0.11**	− 0.13**	− 0.09**

**p < 0.01; *p < 0.05

As shown in Table 4, four variables predict work fatigue in its three dimensions, namely: being exposed to heat and noise most of the time; making efforts to control one’s emotions; requiring constant concentration and workwear (uniforms, suits, or specific clothing). Variables with a more limited effect, since they predict only mental work fatigue, are performing repetitive or monotonous tasks and being exposed to harsh environmental conditions (outdoors, on the street, and so on).

Regression analyses were also performed to identify the effects of the different dimensions of WF on absenteeism, occupational accidents (workplace and commuting), job satisfaction, sleep quality, and health self-assessment variables (see Table 5). It is observed that physical and mental work fatigue impact mental health self-assessment (*β* = 0.30, and *β* = 0.60, respectively). In contrast, no effect of emotional work fatigue on mental health self-assessment is observed, but it does impact work absenteeism (*β* = 0.65). In addition, both physical and mental work fatigue impact sleep quality (*β* = − 0.54, and *β* = − 0.38, respectively).

**Table 5:** Regressions of WF dimensions as predictors of workers’ outcome and quality of life

Pred	Workers’ outcome and quality of life variables							
	GHS	PHS	MHS	ABS	WS	Sleep	WA	CA
	β	β	β	β	β	β	β	β
PH	− 0.15	0.17	0.30*	0.04	0.25	− 0.54**	0.05	− 0.03
ME	− 0.20	0.19	0.60**	0.01	0.31*	− 0.38**	0.04	0.02
EM	− 0.10	− 0.07		0.65**	0.25	− 0.18	0.07	0.01

*Note* GHS = General Health Self-Evaluation; PHS = Physical Health Self-Evaluation; MHS = Mental Health Self-Evaluation; ABS = Absenteeism; WS = Work Satisfaction; Sleep = Sleep quality; WA = Workplace Accidents; and CA = Commuting to Work Accidents. Predictors: PH = Physical WF; ME = Mental WF; and EM = Emotional WF

**p < 0.01; *p < 0.05

## Discussion

The objective of adapting and providing evidence for the validity of the 3D-WFI of Frone and Tidwell [4] for measuring WF in the Chilean working population has been fulfilled. Cronbach’s α and Omega psychometric reliability indices are high, reaching 0.97 and from 0.92 to 0.95 per dimension.

As regards validity, the analyses have shown a Pearson correlation of 0.74 with another international measurement instrument for measuring WF, namely the CIS, by Beurskens et al. [20]. In addition, predictive analyses showed that factors that have traditionally been shown in the literature on WF are significant in explaining WF in its three dimensions in this work, namely: being exposed to heat and noise most of the time; performing a job that requires controlling one’s emotions; permanent concentration; and workwear requirements [8, 9, 14]. Also, in the expected direction, although with a more limited predictive effect, WF of the mental type is additionally explained by two variables: performing repetitive or monotonous tasks and being exposed to complex environmental conditions (on the street, outdoors, etc.).

Overall, the results supported the good fit of the items to the dimensionality proposed by the 3D-WFI scale and indicated that when the items of the structure are kept invariant as a function of sex, the fit indices were satisfactory, except for the parameters of scalar invariance and strict invariance. In this case, partial invariance would be assumed [27], even though the literature has recognized that strict invariance models are often overly restrictive [28]. Therefore, scores may be predominantly comparable between groups, and the change of one unit would be equivalent.

When we sought to identify the effects of WF on aspects related to the functioning of workers, we observed that physical and mental WF have an impact on the self-assessment of their mental health, a relationship that has been observed in previous studies [1, 29]; in addition, our findings indicate that although no effect of emotional fatigue on self-assessment of health is observed, it has a negative impact on absenteeism, as has been observed in nursing staff [30]. Finally, physical and mental WF impact sleep quality, a relationship consistently found in the occupational health literature [11, 31, 32].

### Clinical implications

Work fatigue is a serious problem affecting workers’ performance and safety in their everyday lives. There is increasing attention on fatigue as an intervening variable in occupational accidents, especially in those involving more days of lost time and severity [33, 34]. In this vein, we have confirmed that 3D-WFI may predict important outcomes for workers’ health and organizations, corroborating its condition as a valuable screening tool for researchers and practitioners in organizational settings. In Spanish-speaking countries, a validated tool to assess workers’ fatigue is essential to prevention strategies.

## Limitations

First, the internal consistency obtained for the 3D-WFI are high, and some authors suggest that desirable Cronbach’s alpha values are between 0.70 and 0.90, where values higher than the latter could mean redundancy or duplication [35]. However, when it comes to health assessment instruments, other authors suggest that the desirable minimum should be 0.90 [36]. Likewise, this work uses workers’ self-reports; thus, social desirability could have affected their responses. Not measuring or controlling the participants’ social desirability constitutes a limitation of the present study. Also, using a single, convenient sample might introduce a potential for selection bias. Finally, being a cross-sectional study, causality relationships between work fatigue and associated factors cannot be established.

## Conclusions

The Chilean Spanish version of the 3D-WFI can be reliably and validly applied to the country’s working population in the fields of safety, health and education, and will probably work equally well psychometrically in other areas of national and Latin American production and industries.

## Data Availability

The datasets used and/or analyzed during the current study are available from the corresponding author on reasonable request.
